# 

**Published:** 1899-12

**Authors:** 


					﻿NOTICE,—This JOURNAL and THE INTERNATION-
AL JOURNAL OF SURGERY one year for $1.00 cash,
"to’new subscribers. No out-of-town Checks ac-
cepted unless lOc. is added for cost of cashing.
NOTICE —This JOURNAL and THE INTERNATIONAL
JOURNAL OF SURGERY one year for $1,00 cash, to new
sabscribers. Oat~of-toivn checks not accepted unless 10c. is
added for cost of collection.
NOTICE.—This JOURNAL and THE INTERNATION-
AL JOURNAL OF SURGERY one year for $1.00 cash,
to new subscribers. No out-of-town checks ac-
cepted unless lOc. is added for cost of cashing.
				

